# Prevalence of oral epithelial dysplasia and oral squamous cell carcinoma in Alexandria University Hospital in Egypt: a ten-year retrospective study

**DOI:** 10.1186/s12903-026-08256-z

**Published:** 2026-04-13

**Authors:** Mai M. Saleh, Madonna E. Fawzy, Amira S. Mohamed, Mohamed Mekky, Mona S. Oraby

**Affiliations:** 1 Oral Pathology Department, Faculty of Dentistry, Alexandria University, Alexandria, Egypt; 2 Oral Pathology Department, Faculty of Dentistry, Pharos University, Alexandria, Egypt; 3 Pediatric Dentistry and Dental Public Health Department, Faculty of Dentistry, Alexandria University, Alexandria, Egypt; 4 Oral and Maxillofacial Surgery Department, Faculty of Dentistry, Kafr El-Sheikh University, Kafr El-Sheikh, Egypt; 5 Oral and Maxillofacial Surgery Department, Faculty of Dentistry, Alexandria University, Alexandria, Egypt

**Keywords:** Oral premalignant lesions, Oral squamous cell carcinoma, Oral cancer, Epidemiology and Middle East

## Abstract

**Introduction:**

Oral epithelial dysplasia (OED) is a group of long-term oral mucosal disorders associated with a high risk of malignant transformation. Oral squamous cell carcinoma (OSCC), which represents about 90–95% of oral cancers, can develop from dysplasia in the oral mucosa, which has the potential to be malignant. OSCC still has a poor prognosis because of metastasis and local aggression, even with recent advancements in treatment techniques.

**Aim:**

To determine the prevalence of OED and OSCC based on histopathological examination at Oral Pathology Department in faculty of Dentistry in Alexandria University, Egypt between 2014 and 2023.

**Materials and methods:**

From all patients recorded in Oral Pathology department in faculty of Dentistry in Alexandria University, Egypt between 2014 and 2023, histopathologically proven cases of OED and OSCC have been included. The prevalence of OED and OSCC and its change across the last ten years were assessed. The histopathological grade and some demographic and clinical data for those patients were also investigated.

**Results:**

During the period from 2014 to 2023, 56 cases of OED and 316 OSCC cases were reported based on histopathological examination in the Oral Pathology Department at Faculty of Dentistry, Alexandria University. Most of the OED cases were mild and moderate (each represented 42.86% of OED cases), while severe OED represented 14.28% of OED cases. The majority of OSCC cases (73.73%) were moderately differentiated. Males were more affected than females representing 62.5% of OED cases and 56% of OSCC. The mean age of OED cases was 49.63 years (range = 30, 79) and mean age of OSCC cases was 54.19 years (range = 13, 84).

**Conclusions:**

There was a higher prevalence of OSCC than OED between 2014 and 2023. Both were more prevalent among males and middle age group. The rise in OSCC cases after 2020 emphasize the need for population-based studies and community level approaches for prevention and early diagnosis of OSCC.

## Introduction

Cancer is a significant cause of morbidity and mortality in many countries. According to a global cancer statistic done in 2018, there were around 177,384 deaths and 354,864 new cases of lip and oral cancer recorded worldwide [1]. The age-standardised mortality rates due to lip and oral cancer are higher among the low and low-middle socio-demographic Index regions [2]. Smoking tobacco and smokeless tobacco are the most important risk factors of oral cancer in addition to alcohol and human papillomavirus (HPV) [3].

The term “oral potentially malignant disorders (OPMDs)” refers to a group of asymptomatic clinical lesions associated with tobacco and areca nut consumption that occur before the majority of oral cancer cases. Leukoplakia, erythroplakia, and oral submucous fibrosis, reverse smokers’ palate, actinic keratosis and lupus erythematosus are some clinical presentations of the OPMDs [4]. Although these clinical presentations are considered potentially malignant, the actual diagnosis of oral epithelial dysplasia (OED) or carcinoma requires histopathological confirmation.

OED is a histological correlate for OPMDs. It occurs when cells exhibiting variable degrees of cellular atypia and maturational abnormalities replace a portion of the epithelium’s thickness [5]. It is considered an important predictor of malignant lesion transformation. There have been several attempts to classify OED. WHO proposed a classification system in 2005 that categorizes OED into four groups: mild, moderate, severe dysplasia and carcinoma in situ. This classification depended on the presence of different architectural and cytologic abnormalities detected by light microscopy [5]. Kujan et al. [6] have recently created a 2-tier method that divides OED into low and high risk for malignant transformation.

Histopathological grading of oral epithelial dysplasia remains the principal way to indicate the possibility of malignant transformation. Nevertheless, debates about histopathology as method for diagnosing and classifying OED still stand due to low levels of reproducibility, poor intra- and interobserver agreement, and lack of consensus regarding the risk of malignant transformation depending on histopathology [7]. In order to help with the diagnosis and prognosis of OED lesions, a wide range of new immunohistochemical assays markers of malignant transformation have been suggested; even so, these have not shown to be practical or trustworthy [8]. Some researchers suggested that the combination of dysplasia grading and DNA ploidy analysis will yield a stronger predictive value than either method alone [9, 10].

OED’s carcinogenic potential can vary and be unpredictable [11]. Lesions that exhibit characteristics of both carcinoma-in-situ and severe oral epithelial dysplasia (OED) are more likely to develop into frank malignancies and to have chromosomal abnormalities resembling oral squamous cell carcinoma (OSCC).

OSCC accounts for about 90–95% of oral cancer cases [12]. OSCC may be present clinically as ulcerative or proliferative or ulceroproliferative lesions, but confirmation requires histopathological diagnosis. Depending on how the pathologist grades the tumor’s keratinization, pleomorphism, and mitotic activity, OSCC is categorized by the WHO 2022 as well differentiated (Grade 1), moderately differentiated (Grade 2), and poorly differentiated (Grade 3) [13, 14]. The primary reasons for errors in the diagnosis of histological stages of SCC are inadequate biopsy samples or challenges in diagnosing based on histopathological features using standard hematoxylin-eosin staining [15].

The treatment of OSCC is proper resection and reconstruction in a way that regain patient aesthetics and normal functions of the head and neck region [5]. Even if therapy methods have advanced recently, cases are exposed to recurrence and the prognosis for OSCC remains poor because of metastasis and local aggressiveness [16]. The primary cause of OSCC related mortality is local and regional recurrences. The 5-year survival rate in recurrence-affected individuals is 30% compared to 92% in recurrence-free patients [16, 17].

The literature about the OSCC from the Middle East and North Africa (MENA) is scarce [18]. National cancer reporting systems are not adequate and there is a lack of data on OSCC prevalence in several MENA countries [12, 19]. In Egypt, previous research showed that head and neck cancer (cancer of oral cavity, larynx, and pharynx) represented 17–20% of all cancer [18]. Egypt showed one of the highest overall incidence rates of oropharyngeal cancer among the Middle East Cancer Consortium (MECC) countries [20]. The growing prevalence and late diagnosis increase the burden of the head and neck cancer in Egypt. Deficient data is present concerning prevalence of OED and oral SCC in Egypt. This study aimed to determine the prevalence of OED and oral SCC based on histopathological examination in Oral Pathology department, Faculty of Dentistry Alexandria University, Egypt between 2014 and 2023 and assess histopathological grade and some demographic and clinical data for patients as identified from records held in the Oral Pathology department, Alexandria University, Egypt.

## Materials and methods

### Study design

This study is a retrospective review of patients`records in the Oral Pathology department, Faculty of Dentistry, Alexandria University from 2014 to 2023.

### Ethics and study setting

This study was conducted in accordance with the Declaration of Helsinki. It was approved by the Ethical committee in Faculty of Dentistry, Alexandria University in Egypt (number 0978-09/2024). Informed consent to participate was not obtained from study participants. The need for consent to participate was waived by the Ethical committee in Faculty of Dentistry, Alexandria University as obtaining individual consent is impractical with the retrospective nature of the study and the research poses minimal privacy risk. Data confidentiality and privacy were maintained throughout the study.

The study was conducted in the Oral Pathology department, Faculty of Dentistry, Alexandria University.

### Participants

#### Inclusion criteria

All patients referred to Oral Pathology Department between 2014 and 2023 and had histologically proven OED and OSCC.

#### Exclusion criteria

All patients with oral cavity lesions other than oral epithelial dysplasia and OSCC were excluded, and individuals who have squamous cell carcinoma in head and neck anatomical locations other than the oral cavity.

### Methods

Patient records in Oral Pathology department from 2014 to 2023 were reviewed. Records included biopsies taken at Oral surgery and Maxillofacial department and Oral Medicine and Periodontology department and sent to Oral Pathology department for histopathological examination. Excisional biopsy was taken, histologically examined and double pathologist reviewed. World Health Organization (WHO) 2022 classification was used to classify OED and OSCC [13]. Standard protocols for fixation, embedding tissues in paraffin wax, sectioning, and histological staining techniques, were followed to help in identifying cellular structures accurately. Standardized criteria for slide evaluation and interpretation were put in consideration for accurate diagnosis. All histopathological slides were reviewed again and classified. The documentation for biopsies included patient data (name, age, gender and medical history), location of the lesion and specimen description (description of the tissue including any features or characteristics).

### Variables

Prevalence of OED and OSCC every single year in the period between 2014 and 2023 was assessed. Also, the prevalence of each lesion grade was assessed across the same time period. OED was graded as mild, moderate and severe and OSCC was graded as well-differentiated (WDSCC), moderately differentiated (MDSCC) or poorly differentiated (PDSCC) [13]. The gender and age of the patient were also investigated.

### Statistical analysis

Numbers of cases with mild, moderate and severe OED was assessed and percentage from total number of histopathologically examined cases in Oral Pathology Department each year between (2014–2023) and percentage from OED cases were tabulated. Similarly, the numbers of WDSCC, MDSCC and PDSCC cases and percentage from total number of histopathologically examined cases each year and from OSCC cases were tabulated. Descriptive analysis of gender and age of cases each year was also performed.

## Results

**Fig. 1:**
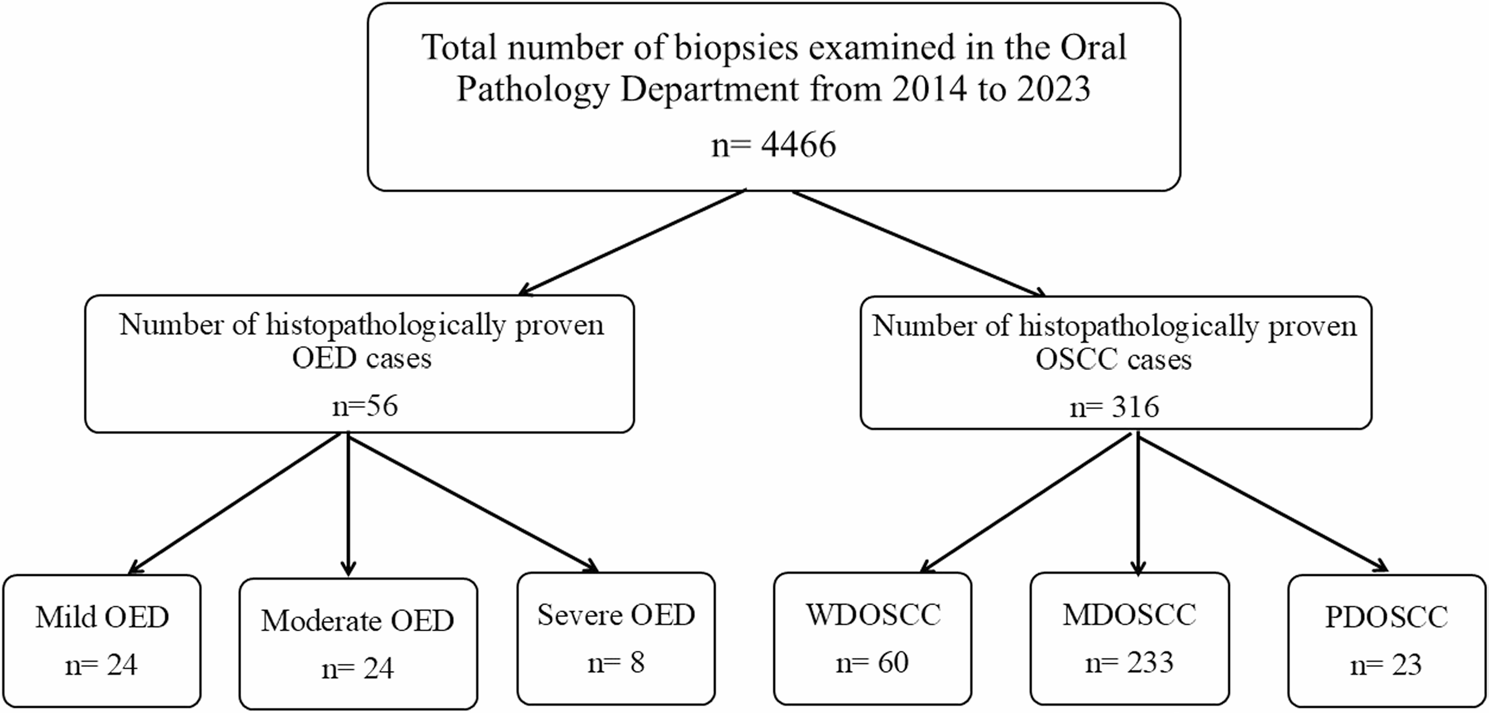
Flow chart of number of biopsies examined in the Oral Pathology Department from 2014 to 2023 and numbers of histopathologically proven OED and OSCC cases

**Fig. 2:**
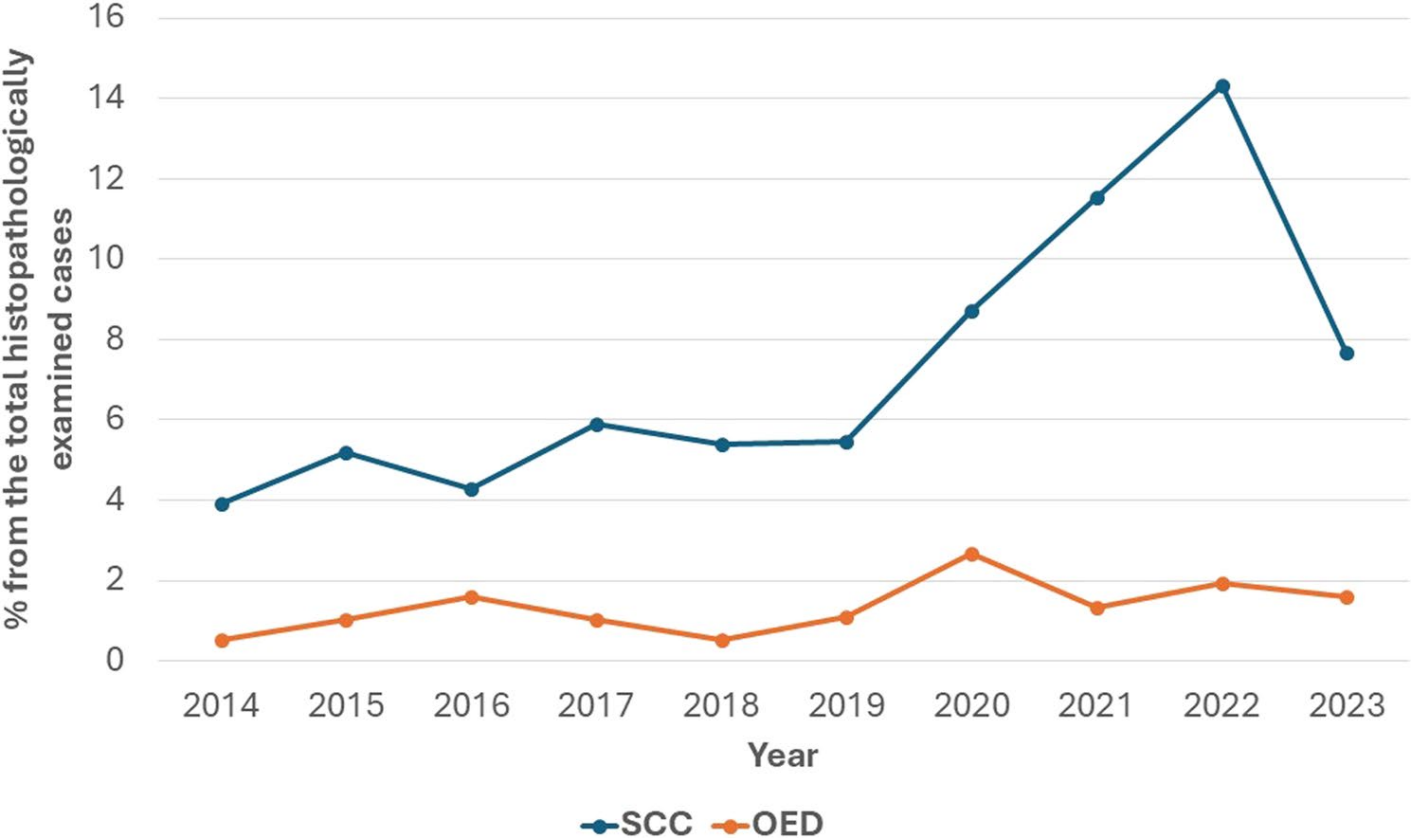
Percentage of histopathologically proven OED and OSCC cases from the total histopathologically examined cases in the Oral Pathology Department, in the period from 2014-2023

During the studied period (2014–2023), excisional biopsies from 4466 cases were histopathologically examined in the Oral Pathology Department, Faculty of Dentistry, Alexandria University. The highest number of examined biopsies (625) was reported in 2023 while the least number (149) was found in 2020. There were 372 cases of histopathologically proven OED and OSCC in the period between 2014 and 2023. Figure 1 is a flow chart showing number of biopsies examined in the Oral Pathology Department from 2014 to 2023 and numbers of histopathologically proven OED and OSCC cases. Across the period 2014–2023, there was a rise in prevalence of OSCC starting from 2020 (Fig. 2).

Records showed that histopathologically proven OED cases was presented clinically as white plaque, red or mixed red and white lesions. Prevalence, gender and age of cases with histopathologically proven OED in the period from 2014 to 2023 are shown in Table 1. 56 cases with OED were reported representing 1.25% of total histopathologically examined cases across the ten-year study period (4466 cases). The lowest prevalence of OED was reported in 2018 (two cases from 390 examined cases, representing 0.51%) while the highest prevalence of OED was reported in 2020 (four cases from 149 examined cases, representing 2.68%).

**Table 1:** Prevalence, gender and age of histopathologically proven OED cases at the Oral Pathology Department, in the period from 2014–2023

Year (Examined cases)		Mild OED	Moderate OED	Severe OED	All OED cases
2014 (382)	*n* (% from examined cases) (% from OED) Males *n* (%) Mean age (Range)	1(0.26) (50) 1 (100) 49	1(0.26) (50) 0 36	0	2 (0.52) (100) 1 (50) 42.5 (36, 49)
2015 (481)	*n* (% from examined cases) (% from OED) Males *n* (%) Mean age (Range)	2 (0.42) (40) 1 (50) 49 (45,53)	1(0.21) (20) 1 (100) 40	2 (0.42) (40) 2 (100) 61.5 (56,67)	5 (1.04) (100) 4 (80) 50.17(40, 67)
2016 (512)	*n* (% from examined cases) (% from OED) Males *n* (%) Mean age (Range)	6 (1.17) (75) 5 (83.33) 58.8 (42,79)	1(0.20) (12.5) 1 (100) 33	1(0.20) (12.5) 0 40	8 (1.56) (100) 6 (75) 43.93 (33, 79)
2017 (576)	*n* (% from examined cases) (% from OED) Males *n* (%) Mean age (Range)	4 (0.69) (66.67) 4 (100) 56.7 (38,75)	2 (0.35) (33.33) 0 72.5 (70,75)	0	6 (1.04) (100) 4 (66.67) 64.6 (38, 75)
2018 (390)	*n* (% from examined cases) (% from OED) Males *n* (%) Mean age (Range)	1(0.26) (50) 1(100) 40	1(0.26) (50) 0 55	0	2 (0.51) (100) 1 (50) 47.5 (40, 55)
2019 (459)	*n* (% from examined cases) (% from OED) Males *n* (%) Mean age (Range)	2 (0.44) (40) 1 (50) 42.5 (30, 55)	2 (0.44) (40) 2 (100) 48 (40, 56)	1 (0.22) (20) 0 44	5 (1.09) (100) 3 (60) 44.83 (30, 56)
2020 (149)	*n* (% from examined cases) (% from OED) Males *n* (%) Mean age (Range)	1(0.67) (25) 0 32	2 (1.34) (50) 1 (50) 36 (32,40)	1(0.67) (25) 1(100) 51	4 (2.68) (100) 2 (50) 39.67 (32, 51)
2021 (529)	*n* (% from examined cases) (% from OED) Males *n* (%) Mean age (Range)	3 (0.57) (42.86) 0 65 (60,70)	4 (0.76) (57.14) 3 (75) 53.5 (40,70)	0	7 (1.32) (100) 3 (42.86) 59.25 (40, 70)
2022 (363)	*n* (% from examined cases) (% from OED) Males *n* (%) Mean age (Range)	1 (0.28) (14.29) 1 (100) 50	5 (1.38) (71.43) 3 (60) 48.4 (45, 55)	1(0.28) (14.29) 0 58	7 (1.93) (100) 4 (57.14) 52.13 (45, 58)
2023 (625)	*n* (% from examined cases) (% from OED) Males *n* (%) Mean age (Range)	3 (0.48) (30) 3 (100) 52.6 (36,62)	5 (0.80) (50) 3 (60) 57.8 (40, 69)	2 (0.32) (20) 1 (50) 53.5 (50, 57)	10 (1.60) (100) 7 (70) 54.63 (36,69)
2014–2023 (4466)	*n* (% from examined cases) (% from OED) Males *n* (%) Mean age (Range)	24 (0.54) (42.86) 17 (70.83) 49.56 (30,79)	24 (0.54) (42.86) 14 (58.33) 48.02 (32,75)	8 (0.18) (14.29) 4 (50) 51.30 (40,67)	56 (1.25) (100) 35 (62.5) 49.63 (30, 79)

There was equal number of cases [24] of mild and moderate OED, each representing 42.86% of OED cases and 0.54% of total histopathologically examined cases. Only 8 cases of severe OED were reported representing 14.29% of OED cases and 0.18% of the total histopathologically examined cases. During the ten-year period, more than half of the OED cases were males (62.5%) which indicates a male to female ratio of 1.67:1. The male predominance among mild OED cases (males represented 70.83%) was relatively higher than that among moderate OED cases (males represented 58.33%). There was equal gender distribution among severe OED cases. Male predominance was found among OED cases in most of the studied years while there was equal gender distribution in 2014, 2018, 2020. In 2021, less males were diagnosed with OED than females (42.86%). The mean age of cases with OED was 49.63 years with a range from 30 to 79 years (Table 1).

Three hundred and sixteen cases of histopathologically diagnosed OSCC were reported to represent 7.08% of total histopathologically examined cases between 2014 and 2023 (Table 2). Across the ten years, OSCC prevalence was the least in 2014 (15 cases from 382 examined cases representing 3.93%) and the highest in 2022 (52 cases from 363 examined cases, 14.33%). The majority of OSCC cases were MDSCC (*n* = 233) representing 73.73% of OSCC cases and 5.22% of total examined cases. Sixty WDSCC cases were reported representing 18.99% of OSCC cases and 1.34% of total examined cases while 23 PDSCC cases were reported representing 7.28% of OSCC cases and 0.52% of total examined cases.

**Table 2:** Prevalence, gender and age of histopathologically proven SCC cases at the Oral Pathology Department, in the period from 2014–2023

Year (Examined cases)		WDSCC	MDSCC	PDSCC	All SCC cases
2014 (382)	*n* (% from examined cases) (% from SCC) Males *n* (%) Mean age (Range)	4 (1.05) (26.67) 3 (75) 56 (35,70)	9 (2.36) (60) 6 (66.67) 49 (42,72)	2 (0.52) (13.33) 1 (50) 50(45,55)	15 (3.93) (100) 10 (66.67) 51.67 (35, 72)
2015 (481)	*n* (% from examined cases) (% from SCC) Males *n* (%) Mean age (Range)	10 (2.08) (40) 3 (30) 59.40 (48,70)	12 (2.49) (48) 10 (83.33) 54.10 (13,60)	3 (0.62) (12) 2 (66.67) 39.60 (27,62)	25 (5.20) (100) 15 (60) 51.03 (13, 70)
2016 (512)	*n* (% from examined cases) (% from SCC) Males *n* (%) Mean age (Range)	5 (0.98) (22.73) 3 (60) 59.80 (47,64)	17 (3.32) (77.27) 15 (88.24) 59.10 (32,75)	0	22 (4.30) (100) 18 (81.82) 59.45 (32, 75)
2017 (576)	*n* (% from examined cases) (% from SCC) Males *n* (%) Mean age (Range)	9 (1.56) (26.47) 5 (55.56) 64 (58,77)	20 (3.47) (58.82) 11(55) 51.9 (43,76)	5 (0.87) (14.71) 5 (100) 59.6 (50,65)	34 (5.90) (100) 21 (61.76) 58.50 (43, 77)
2018 (390)	*n* (% from examined cases) (% from SCC) Males *n* (%) Mean age (Range)	7 (1.79) (33.33) 1(14.29) 45.5 (19,72)	13 (3.33) (61.90) 4 (30.77) 59.10 (40, 82)	1 (0.26) (4.76) 0 49	21 (5.38) (100) 5 (23.81) 51.20 (19, 82)
2019 (459)	*n* (% from examined cases) (% from SCC) Males *n* (%) Mean age (Range)	3 (0.65) (12) 2 (66.67) 50 (40,65)	20 (4.36) (80) 8 (40) 53.2 (36,70)	2 (0.44) (8) 1 (50) 43 (36,50)	25 (5.45) (100) 11(44) 48.73 (36, 70)
2020 (149)	*n* (% from examined cases) (% from SCC) Males *n* (%) Mean age (Range)	2 (1.34) (15.38) 1 (50) 51.5 (33,70)	11 (7.38) (84.62) 8 (72.73) 62.6 (52, 80)	0	13 (8.72) (100) 9 (69.23) 57.05 (33, 80)
2021 (529)	*n* (% from examined cases) (% from SCC) Males *n* (%) Mean age (Range)	8 (1.51) (13.11) 5 (62.50) 61.1(55,70)	51(9.64) (83.61) 23 (45.10) 52.8 (47,84)	2 (0.38) (3.28) 2 (100) 60 (55,65)	61(11.53) (100) 30 (49.18) 57.97 (47, 84)
2022 (363)	*n* (% from examined cases) (% from SCC) Males *n* (%) Mean age (Range)	7 (1.93) (13.46) 4 (57.14) 63.5 (57,77)	40 (11.02) (76.92) 26 (65) 50.7 (22,71)	5 (1.38) (9.62) 3 (60) 55.8 (50,57)	52 (14.33) (100) 33 (63.46) 56.67 (22, 77)
2023 (625)	*n* (% from examined cases) (% from SCC) Males *n* (%) Mean age (Range)	5 ( 0.8) (10.42) 2 (40) 64.6 (50,77)	40 (6.4) (83.33) 21 (52.50) 36.1 (32,72)	3 (0.48) (6.25) 2 (66.67) 60.30 (51, 65)	48 (7.68) (100) 25 (52.08) 53.67 (32, 77)
2014–2023 (4466)	*n* (% from examined cases) (% from SCC) Males *n* (%) Mean age (Range)	60 (1.34) (18.99) 29 (48.33) 57.54 (19, 77)	233 (5.22) (73.73) 132 (56.65) 52.86 (13, 84)	23 (0.52) (7.28) 16 (69.57) 52.16 (27, 65)	316 (7.08) (100) 177 (56.01) 54.19 (13, 84)

Fifty six percent of OSCC cases were males that indicates a male-to-female ratio of 1.27:1. There was male predominance among MDSCC and PDSCC cases (males represented 56.65% and 69.57% of MDSCC and PDSCC cases respectively) while among WDSCC cases, 48.33% were males. Across the ten years, there was male predominance in OSCC cases except for three years (2018, 2019, 2021) where males represented 23.81%, 44% and 49.18% of cases. Patients with OSSC showed a wide age range (13 to 84 years). The mean age was 54.19 years (Table 2).

**Fig. 3:**
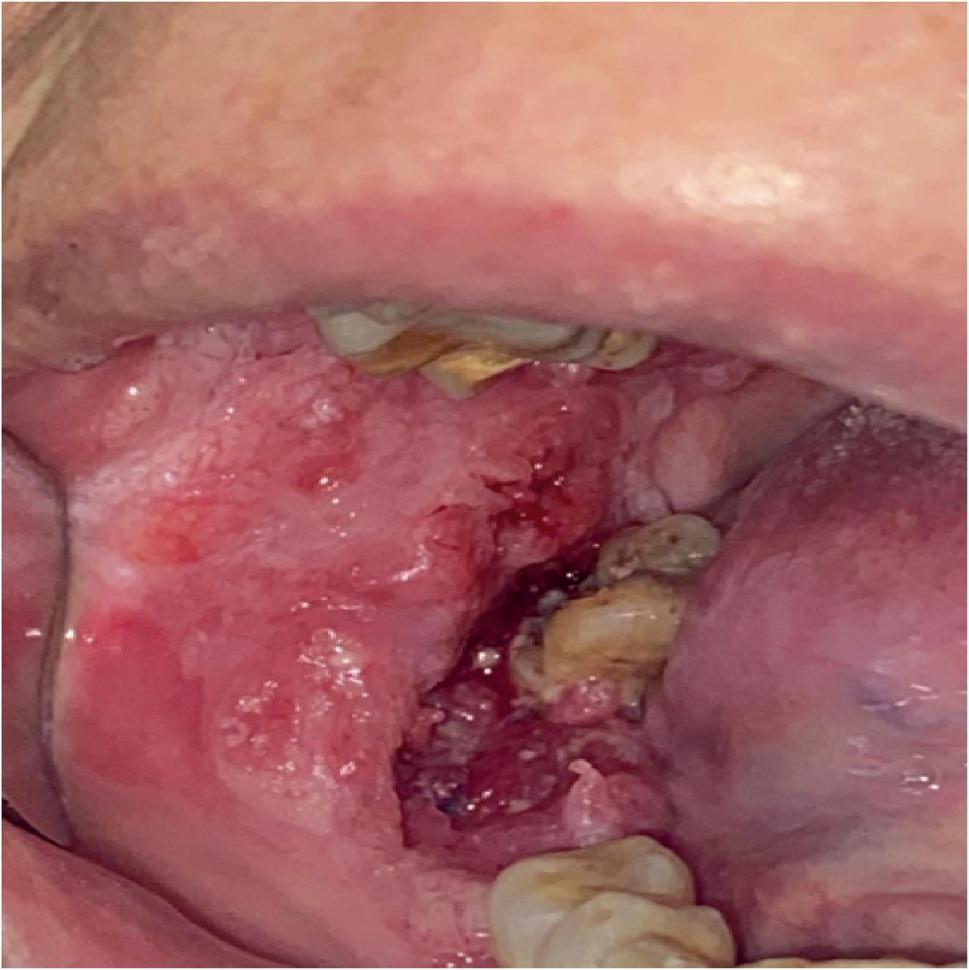
Intraoral image showing OSCC case in the right cheek representing erythroplakic ulcerative lesion

**Fig. 4:**
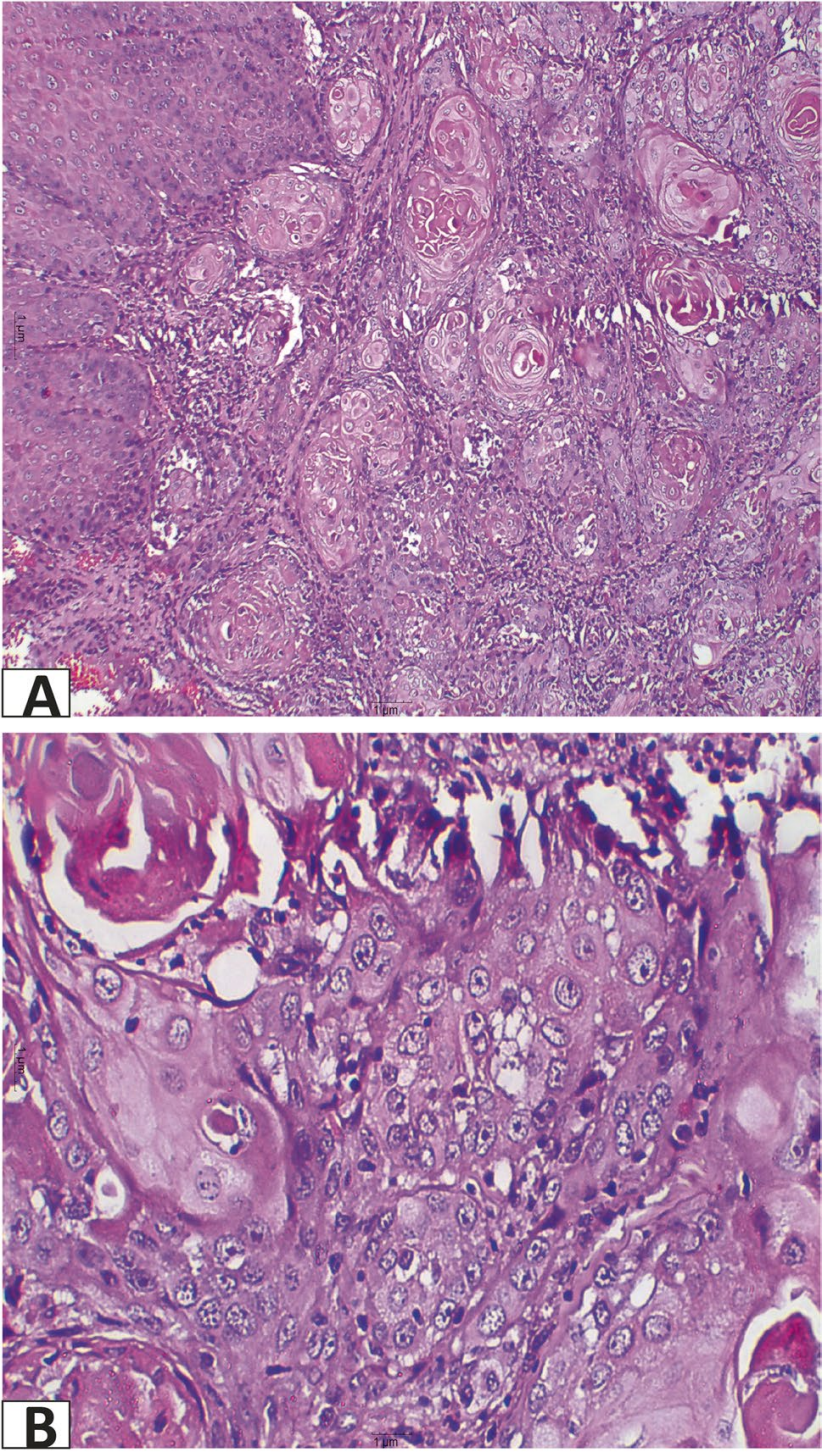
Photomicrograph of well differentiated SCC. **A**: showing malignant epithelial cells invading the underlying connective tissue in the form of epithelial pearls and cell nests (x100). **B**: high power of the previous image showing epithelial pearl with keratin and some malignant criteria such as pleomorphism and hyperchromatism (x400)

**Fig. 5:**
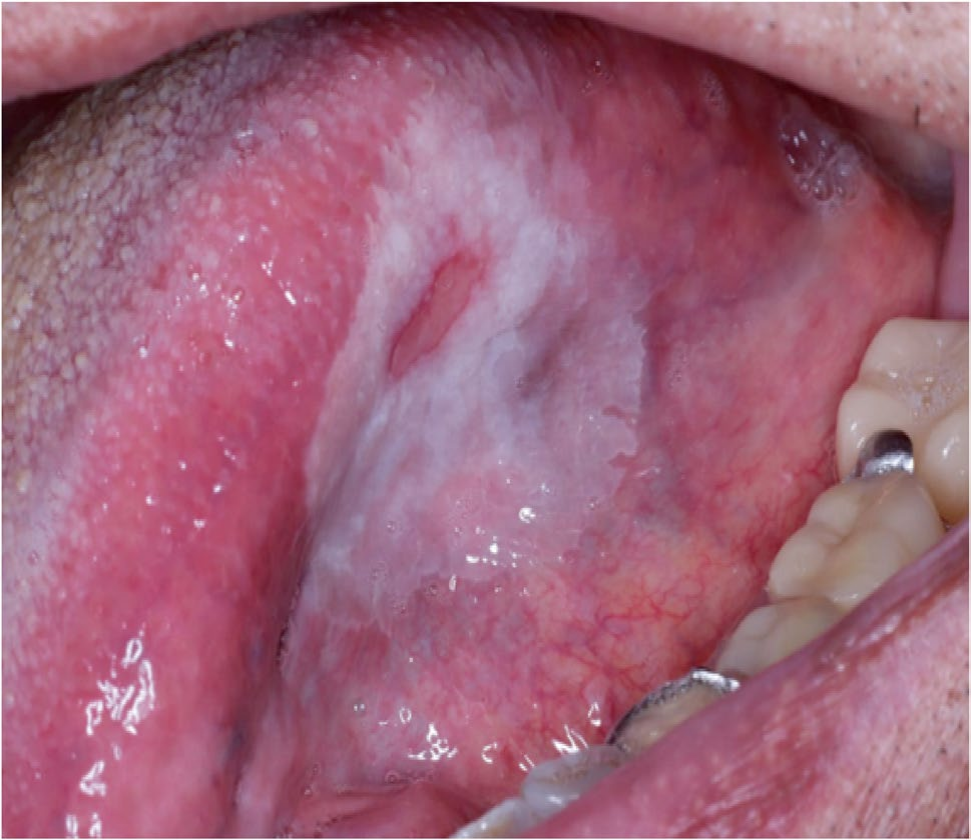
Intraoral image showing white leukoplakic lesion on the left side of the tongue

**Fig. 6:**
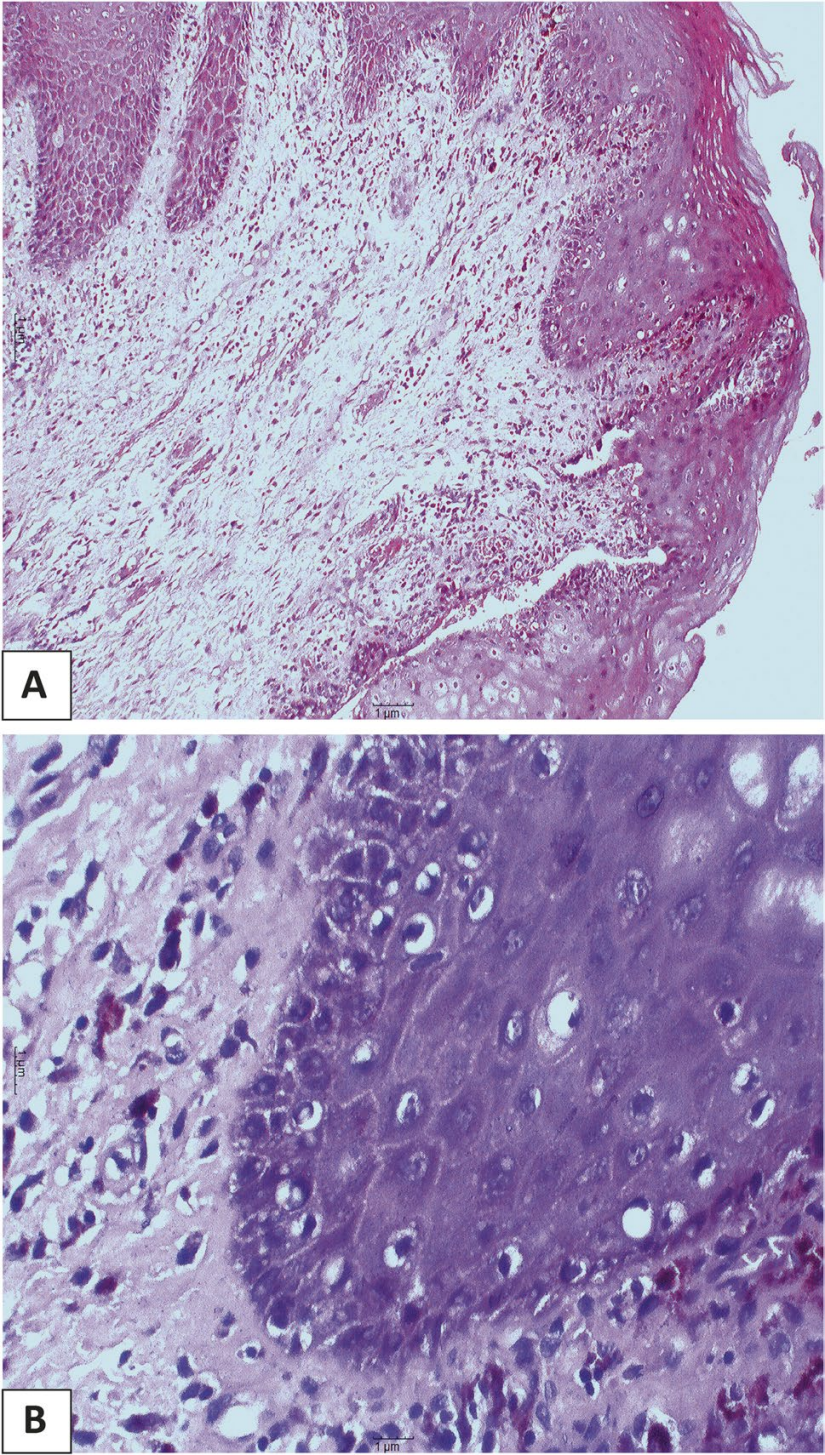
**A**: Photomicrograph showing sever epithelial dysplasia (x100). **B**: high power of the previous image showing malignant criteria of the epithelial cells involving almost all the layers of oral epithelium (x400)

Clinically, the most common site for OED and SCC was tongue. Lesions were also reported in lower lip and buccal mucosa, and to lesser extent in retromolar area, hard palate and upper lip. Figure 3 is a clinical picture of a red ulcerative lesion that was initially suspected as OSCC and subsequently confirmed as OSCC through histopathological evaluation. Figure 4A reveals histological picture of well differentiated OSCC case which shows malignant epithelial cells invading the underlying connective tissue in the form of epithelial pearls and cell nests. Figure 4B shows epithelial pearl with keratin and some malignant criteria such as pleomorphism and hyperchromatism. Figure 5 shows another case clinically presented as white lesion on the lateral side of the tongue. This was initially suspected as OED and subsequently confirmed through histopathological evaluation. Figure 6A reveals histopathological picture of severe epithelial dysplasia with malignant epithelial cells involving almost all the layers of oral epithelium. Figure 6B shows malignant criteria of the epithelial cells such as pleomorphism, hyperchromatism and loss of adhesion between the epithelial cells.

## Discussion

The current study retrospectively assessed OED and OSCC prevalence in a university hospital in Egypt between 2014 and 2023. This study focuses on histopathologically confirmed cases of OED and OSCC. The clinical presentation of these lesions varies and may include leukoplakia, erythroplakia, and ulcerative growths, but only biopsy can determine the final diagnosis. The ten-year prevalence of OED (1.25%) was lower than OSCC prevalence (7.08%) which suggests the need for early histopathological diagnosis of OED before potential malignant transformation. Our findings are comparable to a retrospective study in North India showing mean OED prevalence of 5.71% and mean OSCC prevalence of 9.85% between 2014 and 2018 [21]. However, OSCC prevalence in our study is much higher than an older 20-year retrospective study (1989–2008) in Mexico showing OSCC prevalence increasing from 0.02% to 0.6% at the end of the study [22]. Prevalence of OSCC can greatly vary according to the studied population, time and risk factors.

In 2020, number of histopathologically examined cases in Oral Pathology department in faculty of Dentistry was the lowest across the study period that can reflect the impact of COVID pandemic. People during lockdown might have postponed dental visits to the dental hospital. Number of OSCC cases was also the lowest in 2020 that can be related to COVID impact on cancer diagnosis as shown in other studies [23, 24]. However, relative to the numbers of referred cases, both OED and OSCC showed higher prevalence in 2020 than the previous studied years. OSCC prevalence continued to rise in the subsequent years (2021, 2022) that can be also related to risk factors such as increased smoking and stress during the pandemic [25, 26].

Among OSCC cases, MDSCC was the most prevalent followed by WDSCC. These findings are in agreement with those of Pires et al. who recorded that the majority of OSCC cases were grade 2 moderately differentiated tumors [27]. Similarly, mild and moderate OED were the most prevalent among OED cases. The results were in accordance with the findings published by Kumar et al. [28]. who found that three quarters of patients have low risk dysplastic lesions, and one quarter have high risk dysplasia.

The prevalence of SCC was higher among males which agrees with the general male predominance in head and neck cancers [29] and GLOBOCAN 2020 estimates [30] showing that the age standardised incidence and mortality rates of Oropharyngeal cancer was nearly four times higher among males. Suresh et al. also showed a statistically significant difference between males and females probably due to the prevailing risk factors such as smoking and alcohol drinking among males [31]. Females can be more concerned about the undesirable esthetic impact of smoking such as staining of lips and teeth and bad breath [29].

It is also noteworthy that the current research showed increased prevalence of OSCC among females than males in few years that might reflect the recent increased female exposure to risk factors such as smoking. Thus, the male-to-female ratio of OSCC cases within the ten years (1.27:1) was relatively lower than previous studies showing male-to-female ratio of 2.22:1 [32–34]. High male to-female ratio as high as 6.6: 1 was reported elsewhere [35].

Also, the prevalence of OED was higher among males in accordance with Singh et al. [21]. whereas Pereira et al. who conducted a retrospective study of records over 38 years found that females represented 57.9% of OEDs [36].

The mean age for OSCC was 54 years which is compatible with previous studies reporting mean age for oral cancer of 46.93 [37], 58.62 [35], 60 [34] and 62.3 years-old [27]. The middle age group may represent a period of cumulative exposure to risk factors and the potential onset of age-related cellular changes that can contribute to carcinogenesis. The mean age for OED was a bit younger than that for SCC that can be related to the fact that many SCC lesions are preceded by OED. Developing cancer from dysplastic lesions can occur within two to five years [38].

Our results are consistent with the majority of published material, where the tongue was the most frequent site of OSSC [27, 37]. On the other hand, previous research [39–42] found that the most prevalent location was the buccal mucosa among South Asians. These different findings can reflect the distribution of risk factors of oral cancer. Consumption of smokeless tobacco which is related to oral mucosa is more prevalent in south Asia while smoking manufactured cigarettes and water pipe smoking is more prevalent in Egypt [18, 43].

Caution should be used when interpreting the existing findings in light of the non-representative sample of the Egyptian population. The study had the limitations of secondary data analysis as data on relevant risk factors such as smoking and socioeconomic status was not collected. On the other hand, this study is among very few studies on cancer prevalence in University Hospitals in Egypt and it considered long time period (ten years) which was very dynamic with the COVID breakdown.

The current study shows the need for a more comprehensive records for patients in university hospitals in Egypt. There is also a need for multicenter and population based studies of OED and OSCC prevalence, risk factors and management in Egypt particularly with the rising number of cases [44]. This alarming trend unequivocally emphasizes the pressing need for the immediate implementation of robust, population-based studies. Such studies are crucial for accurately quantifying the true incidence and prevalence of OSCC and identifying emerging risk factors.

Furthermore, this rise in OSCC cases underscores the imperative for developing comprehensive community-level approaches aimed at both prevention and early diagnosis of OSCC. Prevention strategies should encompass public health campaigns raising awareness about risk factors like tobacco and alcohol use, promoting healthy dietary habits, and encouraging regular dental check-ups. For early diagnosis, efforts should focus on enhancing screening programs, training healthcare professionals in recognizing subtle signs of oral lesions, and improving access to diagnostic services, particularly in underserved communities. Although clinical lesions such as leukoplakia or erythroplakia are often the first sign of potential malignancy, only histopathological evaluation confirms the presence of epithelial dysplasia or carcinoma. Early diagnosis can help to decrease the morbidity and mortality and thus burden of the disease.

## Conclusions

This study showed that the ten-year prevalence of OED was lower than that for OSCC. There was a notable surge in OSCC cases observed after 2020. Potential contributing factors could include delayed diagnoses due to the global health events of that period, evolving environmental exposures, or shifts in lifestyle habits.

Both OSCC and OED exhibited a higher prevalence among males, suggesting a gender-specific predisposition that can warrant further investigation into potential behavioural, genetic, or environmental factors. Both OSCC and OED are prevalent among the middle-age group. Population based studies on OED and OSCC prevalence, risk factors and management in Egypt are recommended. Community-level approaches are required for prevention and early diagnosis of OSCC.

## Data Availability

The data that supports the findings of this study are available from the corresponding author upon reasonable request.

## Abbreviations

OPMDs: Oral potentially malignant disorders
OSCC: Oral squamous cell carcinoma
OED: Oral epithelial dysplasia
WDOSCC: Well differentiated oral squamous cell carcinoma
MDOSCC: Moderately differentiated oral squamous cell carcinoma
PDOSCC: Poorly differentiated oral squamous cell carcinoma
